# Characterization of housekeeping genes in zebrafish: male-female differences and effects of tissue type, developmental stage and chemical treatment

**DOI:** 10.1186/1471-2199-9-102

**Published:** 2008-11-12

**Authors:** Amy T McCurley, Gloria V Callard

**Affiliations:** 1 Department of Biology, Boston University, 5 Cummington Street, Boston, MA 02215, USA

## Abstract

**Background:**

Research using the zebrafish model has experienced a rapid growth in recent years. Although real-time reverse transcription PCR (QPCR), normalized to an internal reference ("housekeeping") gene, is a frequently used method for quantifying gene expression changes in zebrafish, many commonly used housekeeping genes are known to vary with experimental conditions. To identify housekeeping genes that are stably expressed under different experimental conditions, and thus suitable as normalizers for QPCR in zebrafish, the present study evaluated the expression of eight commonly used housekeeping genes as a function of stage and hormone/toxicant exposure during development, and by tissue type and sex in adult fish.

**Results:**

QPCR analysis was used to quantify mRNA levels of *bactin1, tubulin alpha 1(tuba1), glyceraldehyde-3-phosphate dehydrogenase (gapdh), glucose-6-phosphate dehydrogenase (g6pd), TATA-box binding protein (tbp), beta-2-microglobulin (b2m), elongation factor 1 alpha (elfa)*, and *18s ribosomal RNA (18s) *during development (2 – 120 hr postfertilization, hpf); in different tissue types (brain, eye, liver, heart, muscle, gonads) of adult males and females; and after treatment of embryos/larvae (24 – 96 hpf) with commonly used vehicles for administration and agents that represent known environmental endocrine disruptors. All genes were found to have some degree of variability under the conditions tested here. Rank ordering of expression stability using geNorm analysis identified *18s*, *b2m*, and *elfa *as most stable during development and across tissue types, while *gapdh, tuba1*, and *tpb *were the most variable. Following chemical treatment, *tuba1, bactin1*, and *elfa *were the most stably expressed whereas *tbp, 18s*, and *b2m *were the least stable. Data also revealed sex differences that are gene- and tissue-specific, and treatment effects that are gene-, vehicle- and ligand-specific. When the accuracy of QPCR analysis was tested using different reference genes to measure suppression of *cyp19a1b *by an estrogen receptor antagonist and induction of *cyp1a *by an arylhydrocarbon receptor agonist, the direction and magnitude of effects with stable and unstable genes differed.

**Conclusion:**

This study provides data that can be expected to aid zebrafish researchers in their initial choice of housekeeping genes for future studies, but underlines the importance of further validating housekeeping genes for each new experimental paradigm and fish species.

## Background

Due to their rapid *ex utero *development, optically clear embryos, ease of chemical administration, short generation time and many other advantages, zebrafish (*Danio rerio*) have experienced a rapid growth in popularity as a research model [1]. A PubMed search of zebrafish articles between 1998 and 2008 revealed ~8500 publications, a more than 80% increase over the previous decade. The impetus for expansion of zebrafish research can be ascribed in part to sequencing of the genome and technical advances in manipulating gene functions, but continued development and validation of molecular tools in this model is needed. To measure gene expression changes associated with normal development and physiology, endocrine disruption, toxicology and drug discovery, zebrafish researchers increasingly apply real-time quantitative reverse transcription PCR (QPCR). QPCR has many benefits including fast readout, high sensitivity, reproducibility, and the potential for high throughput as well as accurate quantification [2,3]; however, there are problems associated with its use, including the intrinsic variability of RNA, impurities during RNA extraction, and differences in reverse transcription and PCR efficiencies [4]. It is important, therefore, to apply an accurate method of normalization to control for these errors.

A widely used method for normalization involves the measurement of an internal reference or "housekeeping" gene. Housekeeping gene normalization has the advantage over some other methods in that it takes into account many variables such as enzyme efficiency and RNA quality. The characteristics required of an ideal reference gene should include its stable expression in samples from different subjects, different tissues, across developmental and life stages, and after undergoing experimental treatments. If these requirements are not met, normalization to a varying reference gene could produce erroneous results [4]. Recent findings in mammalian tissues and cell lines reveal that commonly used housekeeping genes such as *bactin1 *and *gapdh *may be inappropriate as internal references because of their variability [4-6]. Additional studies have demonstrated the potential regulation of typically used housekeeping genes under experimental conditions [6-8]. A survey of 100 papers using QPCR in zebrafish shows *bactin1 *as the most popular housekeeping gene with 40 publications. The remaining articles employed *gapdh *(n, 15), *18s *(n, 9), *elfa *(n, 8), *b2m *(n, 1), *g6pd *(n, 1) and other/unlisted (n, 24) as their housekeeping genes. A panel of eight housekeeping genes has been evaluated in fathead minnows [9], but only one other study has compared different housekeeping genes in zebrafish [10] and there remains a need for further validation and characterization under additional conditions.

As part of a program of research in which we are studying genes involved in estrogen biosynthesis, estrogen actions and endocrine disruption in zebrafish [11-13], we observed discrepancies when using different housekeeping genes to normalize QPCR data. The present study was designed to systematically evaluate expressed levels of eight commonly used housekeeping genes as a function of developmental stage and chemical treatment in embryos/larvae, and by tissue type and sex in adult zebrafish. Results reported here show that all genes tested display some degree of variability under the conditions tested, identify those most suitable for studying development, different tissue types and chemical treatments, and illustrate how normalizing with an unstable housekeeping gene can affect apparent experimental outcome.

## Results

### PCR efficiency analysis

**Figure 1:**
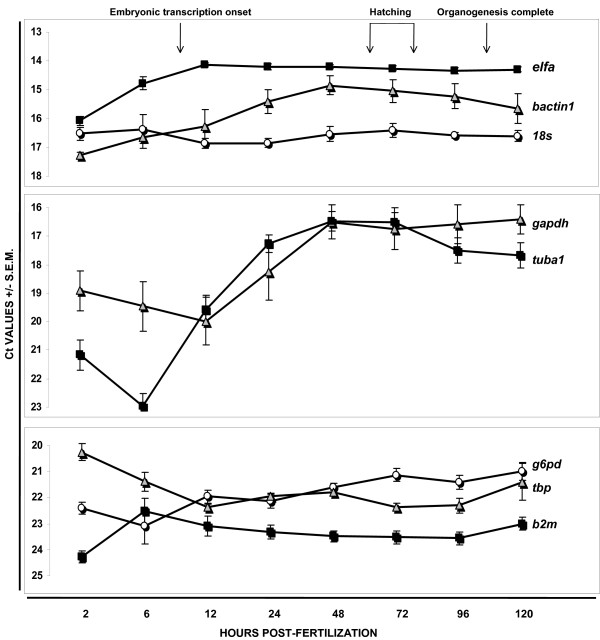
**Housekeeping gene expression during development, as measured by QPCR**. Embryos were collected at timed intervals (2, 6, 12, 24, 48, 72, 96, and 120 hpf) and pooled (50 embryos/pool, 3 pools per time point) for analysis. Ct values represent mean +/- SEM from three biological replicates. Note that the y-axis differs in the three panels to show gene groups based on Ct range during development: (top to bottom) highly expressed genes (*bactin1, elfa, 18s*), genes with highly variable expression *(tuba1, gapdh), g*enes with moderate expression *(tbp, b2m, g6pd)*. All genes showed significant differences across developmental stages by one-way ANOVA p < .05.

Eight housekeeping genes were selected for analysis from commonly used reference genes. Gene names, abbreviations, cellular functions, GenBank accession numbers and primer sequences are listed in Table 1. All primers were optimized for efficiency as follows: A cDNA dilution series (100%, 50%, 20%, 10%, 5%, 2%, and 1%) was developed from embryonic RNA and QPCR was performed on each gene using the dilution series as template. Ct values were exported to *QGene *and efficiency values for each primer pair were determined (see Methods). The dilution series was duplicated using cDNA prepared from adult tissue RNA to verify that similar primer efficiencies were obtained. Using optimized QPCR conditions, all targeted mRNAs were detected during all developmental stages and in all tissue types of both sexes, but Ct values varied, indicating that transcript abundance is gene-, stage-, tissue-, sex- and treatment-related (Tables 2 &3; Fig. 1).

**Table 1:** Genes selected for expression analysis

*Gene Name*	*Gene Symbol*	*Cellular Function*	*Primer Sequence (5'-3')*	*Reference*	*Accession #*
*bactin1*	*bactin1*	Cytoskeleton	F) CGAGCAGGAGATGGGAACC	14	AF057040
			R) CAACGGAAACGCTCATTGC		

*tubulin, alpha 1*	*tuba1*	Cytoskeleton	F) CCTGCTGGGAACTGTATTGT	*	AF029250
			R) TCAATGAGTTCCTTGCCAAT		

*glyceraldehyde-3-phosphate dehydrogenase*	*gapdh*	Glycolysis enzyme	F) GTGGAGTCTACTGGTGTCTTC	15	BC083506
			R) GTGCAGGAGGCATTGCTTACA		

*glucose-6-phosphate dehydrogenase*	*g6pd*	Glycolysis enzyme	F) GTCCCGAAAGGCTCCACTC	9	BM182602
			R) CCTCCGCTTTCCTCTC		

*TATA-box-binding protein*	*tbp*	Transcription	F) CGGTGGATCCTGCGAATTA	*	NM_200096
			R) TGACAGGTTATGAAGCAAAACAACA		

*beta-2-microglobulin*	*b2m*	Major histocompatibility complex	F) GCCTTCACCCCAGAGAAAGG	*	BC062841
			R) GCGGTTGGGATTTACATGTTG		

*elongation factor 1-alpha*	*elfa*	Translation	F) CTTCTCAGGCTGACTGTGC	16	AY422992
			R) CCGCTAGCATTACCCTCC		

*18s ribosomal RNA*	*18s*	Ribosome subunit	F) TCGCTAGTTGGCATCGTTTATG	17	BX296557
			R) CGGAGGTTCGAAGACGATCA		

*cytochrome P450, family 19, subfamily A, polypeptide 1b*	*cyp19a1b*	*Steroid biosynthesis*	F) AAAGAGTTACTAATAAAGATCCACCGGTAT	13	AF226619
			R) TCCACAAGCTTTCCCATTTCA		

cytochrome P450, family 1, subfamily A	*cyp1a*	*Xenobiotic metabolism*	F) GCATTACGATACGTTCGATAAGGAC	18	NM_131879
			R) GCTCCGAATAGGTCATTGACGAT		

The first 8 were candidate housekeeping genes. c*yp19a1b *and *cyp1a*, targets of ER and AhR mediated signal transduction, respectively, were used to tests effects of normalization on apparent gene expression. Asterisks (*) indicate primers designed in this study.

**Table 2:** Housekeeping gene expression in different tissues of adult male (M) and female (F) zebrafish, as measured by QPCR

**Ct Values +/- S.E.M.**												
	**EYE**		**BRAIN**		**HEART**		**LIVER**		**MUSCLE**		**GONAD**	

**GENE**	**M**	**F**	**M**	**F**	**M**	**F**	**M**	**F**	**M**	**F**	**M**	**F**

***bactin1***	16.2 ± 0.45	16.4 ± 0.26	16.4 ± 0.33	16.4 ± 0.46	17.5 ± 0.33	**16.7 ± 0.57**	18.2 ± 0.42	17.7 ± 0.30	17.7 ± 0.58	**16.8 ± 0.51**	16.2 ± 0.29	**14.2 ± 0.24**

***tuba1***	23.2 ± 0.19	23.1 ± 0.17	22.2 ± 0.23	22.3 ± 0.18	22.3 ± 0.29	23.5 ± 0.43	27.8 ± 0.28	**29.2 ± 0.27**	27.0 ± 0.40	**25.4 ± 0.18**	21.2 ± 0.30	21.4 ± 0.85

***gapdh***	18.4 ± 0.33	**17.8 ± 0.58**	22.6 ± 0.98	**23.6 ± 0.84**	15.0 ± 0.35	15.2 ± 0.53	15.8 ± 0.62	**16.8 ± 0.62**	15.4 ± 0.07	14.8 ± 0.34	20.1 ± 0.78	**15.0 ± 0.45**

***g6pd***	21.2 ± 0.08	21.6 ± 0.17	21.8 ± 0.11	22.0 ± 0.38	22.2 ± 0.07	**21.4 ± 0.19**	20.3 ± 0.20	21.0 ± 0.12	25.1 ± 0.37	**23.3 ± 0.11**	20.4 ± 0.10	**19.1 ± 0.16**

***tbp***	22.1 ± 0.24	22.6 ± 0.25	21.9 ± 0.19	21.7 ± 0.17	24.1 ± 0.26	**22.2 ± 0.29**	24.0 ± 0.16	23.7 ± 0.08	24.6 ± 0.19	**20.6 ± 0.16**	20.2 ± 0.03	**16.5 ± 0.15**

***b2m***	17.6 ± 0.05	**16.9 ± 0.03**	18.2 ± 0.16	**17.1 ± 0.01**	15.9 ± 0.14	15.8 ± 0.04	16.1 ± 0.01	16.7 ± 0.01	17.4 ± 0.07	16.7 ± 0.29	16.0 ± 0.06	**18.4 ± 0.22**

***elfa***	15.2 ± 0.08	15.3 ± 0.02	15.7 ± 0.02	15.7 ± 0.27	14.8 ± 0.05	14.9 ± 0.18	14.3 ± 0.08	14.2 ± 0.04	16.7 ± 0.11	**16.0 ± 0.04**	14.6 ± 0.09	14.1 ± 0.03

***18s***	16.6± 0.07	16.6 ± 0.08	16.7 ± 0.05	16.9 ± 0.05	17.6 ± 0.13	17.0 ± 0.06	16.5 ± 0.08	16.4 ± 0.11	17.3 ± 0.10	16.8 ± 0.08	16.6 ± 0.07	17.0 ± 0.34

Values represent Ct values (mean +/- SEM) of tissues of each type pooled by sex (3 independent pools per tissue type/sex/5 fish per pool). For details, see Methods and Results. Boldface indicates significant difference between male and female of the same tissue by t-test p < .05. All genes showed significant differences across tissue types by one-way ANOVA p < .05.

**Table 3:** Housekeeping gene expression following vehicle/hormone/toxicant treatment from 24–96 hpf, as measured by QPCR

**Ct values +/- S.E.M.**								
**GENE**	**CONTROL**	**ETOH**	**DMSO**	**0.1 μM E2**	**1 μM T**	**10 μM ICI**	**10 nM BNF**	**1 nM TCDD**

***bactin1***	15.1 ± 0.07	14.9 ± 0.01	15.2 ± 0.12	15.0 ± 0.03	14.7 ± 0.08	15.1 ± 0.05	15.5 ± 0.19	15.1 ± 0.02
***tuba1***	19.9 ± 0.11	19.5 ± 0.08	19.6 ± 0.08	20.0 ± 0.13	19.3 ± 0.08	19.8 ± 0.04	19.7 ± 0.05	19.5 ± 0.08
***gapdh***	16.8 ± 0.12	16.1 ± 0.20*	16.0 ± 0.10*	15.9 ± 0.02	15.9 ± 0.07	15.8 ± 0.03	15.9 ± 0.11	16.1 ± 0.06
***g6pd***	21.4 ± 0.11	20.7 ± 0.04*	20.8 ± 0.03	**21.5 ± 0.06**	20.8 ± 0.01	20.8 ± 0.03	21.4 ± 0.16	21.1 ± 0.07
***tbp***	20.4 ± 0.53	21.6 ± 0.14	20.6 ± 0.31	21.4 ± 0.05	21.9 ± 0.15	**21.9 ± 0.06**	22.1 ± 0.13	**22.3 ± 0.25**
***b2m***	23.8 ± 0.16	23.0 ± 0.13	22.9 ± 0.23	23.6 ± 0.10	**24.3 ± 0.43**	**24.0 ± 0.44**	**24.5 ± 0.30**	**24.2 ± 0.16**
***elfa***	14.6 ± 0.12	14.2 ± 0.01*	14.2 ± 0.01*	14.2 ± 0.05	14.4 ± 0.12	14.3 ± 0.05	14.4 ± 0.07	14.3 ± 0.03
***18s***	16.2 ± 0.02	16.3 ± 0.08	16.8 ± 0.18*	**16.3 ± 0.03**	**16.3 ± 0.03**	**16.3 ± 0.15**	**16.3 ± 0.02**	16.9 ± 0.09

Values represent Ct values (mean +/- SEM) from 3 biological replicates (50 embryos/larvae per pool, 3 pools per treatment group). For details, see Methods and Results. Asterisks (*) indicate significant difference between vehicle [dimethyl sulfoxide (DMSO), ethanol (EtOH)] and control (control = untreated; p < .05). **Boldface **indicates significant difference between treatment [17β-estradiol (E2), testosterone (T), ICI 182,780 (ICI), β-napthaflavone (BNF), tetrachlodibenzo-p-dioxin (TCDD)] and DMSO (p < .05).

### Expression levels of housekeeping genes by sex and tissue type in adult zebrafish

To assess housekeeping gene expression by sex and tissue type, brain, eye, heart, liver, muscle, testes and ovary were collected from adult, reproductively active male and female zebrafish. Tissues were pooled by sex and tissue type (3 pools per sex/tissue type, 5 fish per pool). Mean Ct values for all housekeeping genes in the seven tissues are shown in Table 2. All eight genes showed significant differences in their expression across tissue types when analyzed separately in males and females. The most pronounced variation (~9 Ct) for a given gene (*gapdh*) was found when brain and muscle were compared in females. In addition, when expressed levels of a given gene and tissue type were compared in males and females, some significant differences were observed. For example, the expression of *tbp *and *g6pd *in skeletal muscle and heart was significantly higher in females than in males. Significant differences were also observed in five of the eight genes (*bactin1, gapdh, g6pd, tbp*, and *b2m*) when ovaries and testes were compared. Whether male-female differences in housekeeping gene expression can be translated to individual differences within each tissue pool is a question for future studies.

### Housekeeping gene expression during development

For each of the eight housekeeping genes, transcript abundance was measured in 3 independent embryo/larval pools (50/pool) collected at timed intervals from 2 – 120 hours post fertilization (hpf). Expression levels during development, represented as mean Ct values, are shown in Figure 1. The genes segregated into three categories based on transcript abundance: *elfa, bactin1, and 18s *were highly expressed (Ct < 18), *g6pd, tpb*, and *b2m *were expressed at a moderate level (Ct 20–25), and *tuba1 *and *gapdh *showed highly variable expression levels (Ct 16–23). Each of the eight genes showed significant differences in expression over the developmental time course as determined by ANOVA. However the magnitude of change over the course of development ranged from less than 0.5 Ct (*18s*) to more than 6 Cts (*tuba1*). Generally, the variability in expression was greatest between 2 and 24 hpf and subsequently stabilized.

### The effect of vehicle/hormone/toxicant treatment on housekeeping gene expression in embryos/larvae

To determine whether variation in housekeeping gene expression is affected by chemical treatment, pooled zebrafish embryos/larvae (3 independent pools per treatment type) were exposed between 24–96 hpf to commonly used vehicles for administration and agents that represent known environmental endocrine disruptors [11-13]. Shown in Table 3 are the mean expression levels (Ct values) of each gene in embryos treated with vehicle (DMSO or EtOH), an estrogen receptor (ER) agonist (17β-estradiol, E2), an ER antagonist (ICI 180,172, ICI), an aromatizable androgen/androgen receptor agonist (testosterone, T) and aryl hydrocarbon receptor (AhR) agonists (β-napthaflavone, TCDD). In our laboratory, exposure to these chemicals at the doses used affects ER- or AhR-mediated target gene effects (respectively, *cyp19a1b *and *cyp1a*) without a general toxic response [11-13]. All eight genes tested revealed significant differences in expression levels across treatment groups. Of particular interest are the changes seen with the two vehicles. Both DMSO and EtOH significantly altered expression of three of eight measured genes. Of these, *gapdh *and *elfa *expression increased with DMSO and EtOH when compared to untreated controls, but *g6pd *and *18s *were differentially affected by vehicle treatment. Also, TCDD, a potent toxicant, strongly suppressed expression of one gene (tbp, ~2 Cts), whereas hormonal estrogen (E2) had modest effects on two genes (*g6pd, 18s*). Interestingly, the pattern of effects with T, an aromatizable androgen, that markedly upregulates estrogen responsive genes at the doses used [11], differed from that of E2. This suggests that T effects are ER independent. The overall variability in expression levels of the eight genes following treatment of embryos/larvae was less than that seen when the same mRNAs were measured in different tissue types of adult fish or during the course of development (compare Table 3 with Table 2 and Figure 1). It is noteworthy here that all Ct values had low SEMs, signifying low sample-to-sample biological and technical variation within a given experimental condition.

### Expression stability of housekeeping genes

**Figure 2:**
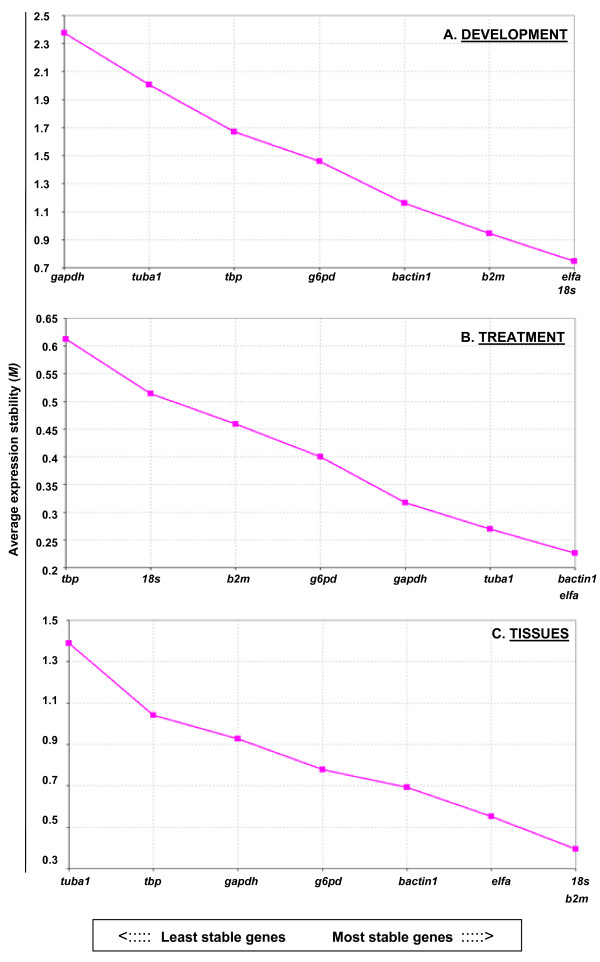
**Expression stability of housekeeping genes**. Results of QPCR analyses from different (A) stages of development (Figure 1); (B) treatment conditions (Table 3); and (C) tissue types (Table 2, male-female data combined) were used to calculate stability using geNorm. For details see Methods and Results. The relative M-values (y-axis) are defined as a measure of gene expression stability, with an increasing M-value correlating with less stability. Note that the range of M-values (high to low), indicating the degree of variability between the least and most stable genes, differed for each of the three conditions: development (3.4-fold), treatment (3-fold), and tissue distribution (4.5-fold).

The relative expression levels of the eight housekeeping genes were calculated for the developmental series, treatment series, and tissue panel (males and females combined). geNorm software was then used to compute the expression stability values (M) for each gene where a lower M value corresponds to more stable gene expression. *18s, elfa*, and *b2m *were found to have the most stable gene expression during development (Figure 2A). Following hormone/toxicant treatment *elfa, bactin1*, and *tuba1 *were the most stably expressed genes (Figure 2B). As with development, *18s, b2m*, and *elfa *also showed the most stable expression across tissue types (Figure 2C). Despite significant sex differences in expressed levels of certain genes and tissue types, the order of gene stability by geNorm analysis varied little when males and females were plotted separately (data not shown).

### Effect of using different housekeeping genes to normalize genes of interest

**Figure 3:**
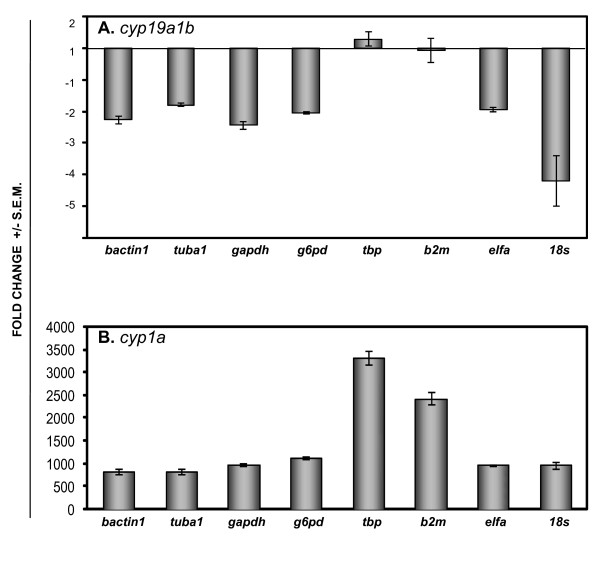
**Effects of normalization with different reference genes on expression of (A) *cyp19a1b *and (B) *cyp1a *in zebrafish embryos following exposure to ICI 180,172 (10 μM) and TCDD (1 nM), respectively**. The results are represented as fold-change (mean ± SEM) compared to the respective DMSO treated controls. c*yp19a1b *(mean Ct value: DMSO = 27.3 ± 0.07; ICI = 28.7 ± 0.02) and *cyp1a *(mean Ct value: DMSO = 25.7 ± 0.05; TCDD = 15.7 ± 0.01) were measured by QPCR as described in Methods. Treatment groups are those described in Methods and Results (Table 3; 50 embryos/pool; 3 biological replicates).

To test the accuracy of QPCR results after normalization with different housekeeping genes, *cytochrome P450 19a1b *(*cyp19a1b*) was selected because it is an estrogen responsive gene that displays a modest downregulation of constitutive expression when embryos are treated with the ER antagonist ICI, as measured by standard RT-PCR/Southern transfer hybridization [12]. As shown in Figure 3, when the expression levels were normalized to housekeeping genes unaffected by ICI treatment (*bactin1, tuba1, gapdh, g6pd, elfa*, see Table 3), *cyp19a1b *expression was down-regulated approximately 2-fold as expected. The modest effect of ICI treatment on *18s *expression however, resulted in an exaggerated down-regulation of *cyp19a1b *(~4 fold) when *18s *was used for normalization. In contrast, ICI had no apparent effect on *cyp19a1b *expression when the data were normalized to those housekeeping genes down-regulated by ICI (*tbp, b2m*). The variation in expression levels due to normalization could not be accounted for by variations in the Ct values of the target gene (see legend for Figure 3). To further test effects of different housekeeping genes, a second target gene, *cytochrome P450 1a *(*cyp1a*), was chosen. *cyp1a *is robustly upregulated by TCDD acting through the AhR, as measured by RT-PCR/Southern transfer [14-18]. When the expression levels were normalized to housekeeping genes unaffected by TCDD treatment (*bactin1, tuba1, gapdh, g6pd, elfa, 18s*) *cyp1a *expression was up-regulated 800 to 1000-fold as expected. In contrast, when *cyp1a *mRNA levels were normalized to housekeeping genes down-regulated by TCDD (*tbp, b2m*) the up-regulation was greatly overstated (2400- to 3300-fold).

## Discussion

There are now numerous reports describing the unreliability of commonly used housekeeping genes for the normalization of QPCR data (see Introduction). The expectation of identifying an "ideal" housekeeping gene that is stably expressed under all conditions was not met in this study. Rather, all eight housekeeping genes evaluated here show unstable expression under more than one experimental condition. Nonetheless, the degree of instability is important to note. We find that *gapdh *is one of the most unstable and *elfa *and *18s *among the most stable genes during development (2 – 120 hpf) and across tissue types. Our results are consistent with the earlier zebrafish study in which five of our eight housekeeping genes were measured between 2 and 72 hpf and in pooled male-female tissues [10]. Based on the agents tested in our treatment series, however, we would eliminate *18s *as a suitable housekeeping gene. Instead, our choices for studies using zebrafish embryos to test hormones and endocrine disrupting chemicals that interact with ER and AhR l would be *elfa *and *bactin1*.

It is important to note that the rank order of gene stability reported in the present study cannot be generalized, without further testing, to other fish species, tissue types, life stages, or treatment paradigms. An examination of the fish literature reveals many contradictory findings [cited in [9]]. To illustrate, a QPCR study of eight housekeeping genes in adult fathead minnows reported that 21 d exposure to ethinyl E2 (EE2) markedly suppresses hepatic expression of four genes, including *elfa, bactin1, gapdh *and *g6pd *(but not *18s*) [9]. A similar study in adult medaka also found a suppression of *bactin1 *expression in the liver with a 21 d exposure to EE2 but in contrast found the EE2 exposure increased hepatic expression of *gapdh *[19]. In zebrafish embryos, on the other hand, we find that a 3 d exposure to E2 suppresses *g6pd*, upregulates *18s *but has no effect on *elfa *or *bactin1*. Similarly, results showing E2 and EE2 effects on *gadph *and *bactin1 *expression in zebrafish by microarray analysis are inconsistent with our QPCR results, indicating that it may be incautious to compare findings using different methods of mRNA analysis [20,21].

Although changes in housekeeping gene expression in response to EtOH, DMSO or low doses of chemicals appear small, even small differences can add significant error to target gene expression during normalization. The example of *cyp19a1b *expression following ICI treatment demonstrates the impact on a modest gene response. When housekeeping genes affected by ICI are used for normalization the expected down-regulation is negated. If this situation were to occur when testing a novel chemical, or when using QPCR to verify results of microarray analysis, a real effect could be overlooked. In the case of *cyp1a*, a gene that is robustly induced by AhR ligands, a difference in housekeeping gene expression results in a more than 3-fold exaggeration in upregulation. This kind of overstatement could have real implications for data interpretation, for example, when comparing dose-response characteristics of different chemical agents, or when screening environmental samples for bioactivity. To avoid unforeseen errors in normalization, for example, by the presence of unknown agents in complex mixtures that affect reference gene expression, the stability of the chosen housekeeping gene can be routinely monitored by recording changes in Ct values.

Given that many of the classical reference genes have proven unreliable [22-28], alternative normalization strategies have been proposed. One typical approach is to normalize to total RNA levels. While this avoids the difficulties of selecting and validating a housekeeping gene, the shortcoming of this method is that it does not control for errors introduced at the reverse transcription step of PCR reactions. In addition, it primarily measures ribosomal RNA (rRNA) whereas qPCR aims to determine mRNA expression. Furthermore, normalization for total RNA assumes that the rRNA: mRNA ratio is the same in all groups, which might not always be the case [2,29,30]. Finally, the high abundance of rRNA compared to mRNA makes it difficult to subtract the baseline value in qPCR analysis [31,32]. For the same reasons, markers of rRNA such as 18s or 28S rRNA might be suboptimal as normalization factors in many settings [2,32,33]. Also, as our data shows, it cannot be assumed that rRNAs are stably expressed under all conditions.

Another promising method for normalization is the use of statistical software to determine the most stably expressed gene [31]. By using the geometric average of multiple control genes, geNorm software provides accurate normalization of qPCR data [34]. When geNorm was applied in this study, the recommended housekeeping genes were indeed those with minimal Ct changes and overall stable expression. Other statistical programs (BestKeeper, Norm Finder) have been developed to determine the most appropriate reference gene for a given experimental condition [31,35]. They use different algorithms to analyze the variation in the expression of reference genes, which could result in different recommendations for the most suitable reference gene. The disadvantage of using statistical programs is that considerable effort and cost is expended to generate data for analysis, but similar constraints apply to any strategy that requires the validation of multiple housekeeping genes.

The intent of this study was to provide a database that helps zebrafish researchers to identify a shortlist of candidate housekeeping genes for specific experiments. For example, although it has been a relatively popular housekeeping gene for zebrafish research, *gapdh *clearly has large variability in its expression under all experimental conditions tested in our study and so would not be recommended for normalization. Studies by Tang *et al *in zebrafish [10] and Filby and Tyler in fathead minnows [9] also found *gapdh *to be unsuitable for data normalization. The gene with the least variability across all the conditions assessed in this study was *elfa *and so may be an appropriate initial selection for normalization.

## Conclusion

All eight housekeeping genes tested were found to have some degree of variability under the conditions tested here, but genes most suitable as normalizers during development, across tissue types, and in chemical treatment experiments were identified. The gene with a low degree of variability across all conditions was *elfa*, whereas *gapdh *was unstable under all conditions. Results of this study are intended to guide zebrafish researchers with initial selection of a reference gene, but underline the importance of accurate housekeeping gene validation for each new experimental paradigm.

## Methods

### Zebrafish and treatments

Wild type adult male and female zebrafish, *Danio rerio*, were obtained from a commercial supplier (Ekkwill, Gibsonton, FL) and maintained in 30 gal aquaria at 28°C on a 14:10 light-dark cycle. Fertilized eggs were collected after natural spawning, washed, and distributed into 20 × 100 mm culture plates (Fisher Scientific). Embryos (150 embryos/50 ml egg water) were allowed to develop at 28°C on a 14L:10D cycle [36]. For developmental expression analysis embryos were collected after timed intervals: 2, 6, 12, 24, 48, 72, and 120 hours post-fertilization (hpf), quick-frozen on dry ice, and stored at -70°C until analysis (3 independent embryo pools, 50 embryos per pool, per time point from the same spawning group). For treatment expression analysis embryos were left untreated until 24 hpf and then exposed to 17β-estradiol (E2; 0.1 μM), testosterone (T; 1 μM), ICI 182,780 (ICI; 10 μM; Tocris Bioscience, Ellisville, MO), β-napthaflavone (BNF; 10 nM), or 2,3,7,8, tetrachlodibenzo-p-dioxin (TCDD; 1 nM; Ultra Scientific, N. Kingstown, RI) dissolved in dimethyl sulfoxide (DMSO). All chemicals were obtained from Sigma-Aldrich (St. Louis, MO) unless otherwise noted. Stock solutions of chemicals were added directly to egg water and replaced daily. In addition, embryos were treated with DMSO alone (final concentration, 0.0006%), EtOH alone (final concentration 0.0005%), or left untreated as a control. Embryos were collected at 96 hpf, quick-frozen on dry ice, and stored at -70°C until analysis (3 independent embryo pools per treatment). Treated embryo RNAs were used for both housekeeping gene expression analysis (Table 3) and gene of interest normalization (Figure 2). Tissues (brain, eye, heart, liver, muscle, gonad) were collected from adult male and female zebrafish, pooled by sex (3 pools per tissue type/sex, 5 fish per pool), quick-frozen on dry ice, and stored at -70°C. Adult fish were reproductively active stock from our breeding colony.

### RNA extraction and reverse transcription (RT)

Adult tissues or whole embryos were homogenized in Tri Reagent (Sigma) and total RNA was extracted as previously described [37] and treated with DNase I (Roche, Indianapolis, IN). An aliquot of each extract was used for spectrophotometry to determine RNA quality and concentration. RNA with a 260/280 ratio between 1.95–2.2 and a 260/230 ratio > 1 and < 3 was considered satisfactory and was used in this study. Each RNA extract was assayed in triplicate and an average value was determined. A 1 μg aliquot was taken of each sample and electrophoresed on an agarose gel to confirm quality and concentration. cDNA was synthesized from total RNA (5 μg; 20 μl final reaction volume) with oligo(dT) priming using SuperScript II reverse transcriptase (Invitrogen, Carlsbad, CA) according to the manufacturer's instructions. For analysis of *18s*, reverse transcription was carried out using random primers which results in a lower reaction efficiency. A minimum of two RT reactions were performed for each biological replicate for technical replicate comparison.

### Oligonucleotides

Eight housekeeping genes were selected from commonly used reference genes (Table 1). All oligonucleotide primers were synthesized by Invitrogen. Gene-specific oligonucleotide primers for *tuba1, tbp*, and *b2m *were developed using Primer Express software (Applied Biosystems) and entered into the Real Time PCR Primer Databank . *bactin1 *primers were obtained from the Real Time PCR Primer Databank [14]. Primer sets for all other gene targets were previously published [see Table 1]. All primer sets spanned an exon-exon junction to avoid errors due to contaminating genomic DNA. Primer sets were tested for specificity using standard RT-PCR and zebrafish embryo cDNA as template to verify production of a single band of the predicted size.

### Quantitative real-time PCR

Real time PCR was performed on an ABI Prism 7900 HT sequence detection system (Applied Biosystems) with SYBR green fluorescent label. Samples contained 1× SYBR green master mix, 2–4 pmol of each primer and 0.25 μl RT reaction for a final volume of 10 μl. Samples were run in triplicate in optically clear 384-well plates (Applied Biosystems). Cycling parameters were as follows: 50°C × 2 min, 95°C × 10 min, then 40 cycles of the following 95°C × 15 s, 60°C × 1 min. For each sample a dissociation step was performed at 95°C × 15 s, 60°C × 15 s, and 95°C × 15 s at the end of the amplication phase to identify a single, specific melting temperature for each primer set. PCR was performed twice on each sample for a minimum of 36 data sets generated for each sample/gene combination (3 biological replicates × 2 RT reactions × 2 PCR runs × 3 reactions per PCR run).

### Data Analysis

Data generated by real-time PCR were compiled and collected using SDS 2.2 software (Applied Biosystems). Data were exported to *QGene *to determine the PCR amplification efficiency (*E*) for each primer pair where *E *= 10^(-1/slope) ^as determined by linear regression analysis of a dilution series of reactions [[38]; see Results]. All amplifications had a PCR efficiency value between 1.9 and 2.2. To normalize data for geNorm analysis the efficiency of each primer pair (*E*), together with the Ct values, was used to calculate a relative gene expression value for each transcript using the equation E ^ΔCt(Min Ct-Ct sample) ^where Min Ct is the lowest Ct value for the primer pair and Ct sample is the Ct value for each amplification [10,34]. The Ct is defined as the number of cycles needed for the fluorescence to reach a specific threshold level of detection and is inversely correlated with the amount of template present in the reaction [39]. The relative stability of the eight reference genes was then calculated using geNorm [34]. This program evaluates a gene expression stability measure (M) for each reference gene by calculating pair-wise variations with all other control genes and ranks them in order of increasing expression stability. Statistical analysis of Ct value differences was performed using the Sigma-Stat 3.5 package (Aspire Software, Leesburg, VA). Data were analyzed by one-way analysis of variance (ANOVA) followed by the Tukey method for pair-wise multiple comparisons. Student's *t*-test was used to compare differences in mean Ct values between male and female tissues. Student's t-test was also used to determine significant differences in expression following chemical treatment. Vehicle (DMSO, EtOH) was compared to untreated and all other chemicals (E2, T, ICI, BNF, TCDD) were compared to the vehicle of preparation (DMSO). Significance was set at *P *< 0.05.

## Abbreviations

PCR: polymerase chain reaction; RNA: ribonucleic acid; Ct: cycle threshold; ANOVA: analysis of variance; S.E.M: standard error of the mean; EtOH: ethanol

## Authors' contributions

ATM conceived of the study, has been responsible for all the experimental work, and has been involved in drafting and revising the manuscript. GVC participated as a supervisor in study design and analyses. She has been involved in drafting the manuscript, revising it critically for important intellectual content, and given final approval for the version to be published
